# Diagnostic Yield of Endobronchial Ultrasound-Guided Transbronchial Needle Aspiration Cytological Smears and Cell Blocks: A Single-Institution Experience

**DOI:** 10.3390/medicina54020019

**Published:** 2018-04-18

**Authors:** Marius Žemaitis, Greta Musteikienė, Skaidrius Miliauskas, Darius Pranys, Raimundas Sakalauskas

**Affiliations:** 1 Department of Pulmonology, Medical Academy, Lithuanian University of Health Sciences, A. Mickevičiaus g. 9, 44307 Kaunas, Lithuania; greta.musteikiene@kaunoklinikos.lt (G.M.); skaidrius.miliauskas@kaunoklinikos.lt (S.M.); raimundas.sakalauskas@kaunoklinikos.lt (R.S.); 2 Department of Pathological Anatomy, Medical Academy, Lithuanian University of Health Sciences, A. Mickevičiaus g. 9, 44307 Kaunas, Lithuania; darius.pranys@kaunoklinikos.lt

**Keywords:** endobronchial ultrasound (EBUS), lung cancer, sarcoidosis, cytology, cell blocks

## Abstract

*Background and Objective:* Endobronchial ultrasound (EBUS) is a minimally invasive endobronchial technique, which uses ultrasound along with a bronchoscope to visualize the airway wall and structures that are adjacent to it. Indications for endobronchial ultrasound-guided transbronchial needle aspiration (EBUS-TBNA) are samplings of mediastinal, hilar lymph nodes, and tumors adjacent to airway walls. EBUS-TBNA has been used in our clinic since 2009. The aim of the study is to evaluate the sensitivity, specificity, positive and negative predictive value, and diagnostic accuracy of cytological and histological specimens, and the safety of EBUS-TBNA in an unselected patient population that has been referred to our hospital. *Materials and Methods:* We have retrospectively analyzed the medical documentation of 215 patients who had EBUS-TBNA performed in our clinic from April 2009 to February 2014. *Results:* There were 215 patients who underwent EBUS-TBNA. A total of 296 lymph nodes were sampled. EBUS-TBNA was diagnostic in 176 (81.9%) cases of cytological, 147 (68.4%) cases of histological, and 191 (88.9%) cases of the combined evaluation. In the lung cancer patients, EBUS-TBNA cytology had a sensitivity of 72.9% and histology of 72.9%, and in the sarcoidosis group, it had a cytology of 55.8% and histology of 64.5%. As all positive cytology and histology specimens were assumed to be true positive, specificity and positive predictive value (PPV) were 100%. The sensitivity and diagnostic accuracy was significantly higher when cytology and histology specimens were combined, compared with cytology or histology results evaluated separately (*p* < 0.05) (for lung cancer 84.1% and for sarcoidosis 78.8%). The sensitivity and diagnostic accuracy of EBUS-TBNA procedures increased significantly over time, with increased experience. There were no complications with EBUS-TBNA in our clinical practice. *Conclusions:* EBUS-TBNA had a high diagnostic yield and was safe in the diagnosis of lung cancer and sarcoidosis. It was most informative when cytology and histology were combined. The informative value of EBUS-TBNA histology increased with our experience.

## 1. Introduction

Endobronchial ultrasound-guided transbronchial needle aspiration (EBUS-TBNA) is a minimally-invasive, endobronchial technique that uses ultrasound along with bronchoscopy to visualize the airway wall and structures adjacent to it, and allows real-time guidance in the sampling of mediastinal and hilar lymph nodes, and tumors [1,2].

Mediastinoscopy was the gold standard for invasive mediastinal staging, providing definite tissue diagnosis at 100% specificity and almost 80% sensitivity [3]. Other options included video-assisted thoracoscopic surgery and blind transbronchial needle aspiration. Since 2005, minimally-invasive methods with high diagnostic yields, such as EBUS-TBNA, have been introduced as reasonable alternatives, primarily for lung cancer staging [4,5,6]. Thereafter, EBUS-TBNA use has been extended to the diagnosis of sarcoidosis [7], metastasis from other solid tumors [8], lymphoma [9], and tuberculosis [10].

The majority of the initial clinical trials with EBUS-TBNA were well-designed prospective trials, in reference centers with high expertise, which used highly preselected patient populations, and were enriched by selecting for particular diseases—which probably provided the highest diagnostic yield [11,12]. Nowadays, there is an increasing interest in evaluating the diagnostic value of EBUS-TBNA in daily clinical practice for unselected patient populations [13]. Endobronchial ultrasound (EBUS) was introduced into clinical practice with the dedicated 22-gauge (G) fine needle for aspiration, so the majority of these trials evaluated only the diagnostic yield of cytological specimens from smears [14]. In the current era of the individualized and targeted therapy of lung cancer, precise histological subtyping and detection of molecular alterations on histological specimens are a standard of care [15]. However, the value of the diagnostic yield of histological EBUS-TBNA specimens in daily clinical practice is less clear.

In Lithuania, EBUS was introduced for the first time in the Department of Pulmonology and Immunology of the tertiary Hospital of the Lithuanian University of Health Sciences, in 2009.

The aim of this study is to evaluate the sensitivity, specificity, positive and negative predictive value, diagnostic accuracy of cytological and histological specimens, and the safety of EBUS-TBNA in an unselected patient population, that was referred to our hospital.

## 2. Experimental Section

Patients with mediastinal or hilar lymphadenopathy, who were referred to EBUS-TBNA between April 2009 and February 2014, were enrolled in this retrospective study. A chest computed tomography was routinely performed on all patients, and those with enlarged hilar or mediastinal lymph nodes (nodes with a short-axis diameter greater than 10 mm) were selected for EBUS procedures.

The procedures were performed under conscious sedation, using midazolam and phentanyl, by three different pulmonologists, each with equal training and expertise. A conventional flexible bronchoscopy was performed before all EBUS procedures, to investigate the tracheobronchial tree for intraluminal pathology, using a flexible bronchoscope. Local anesthesia was achieved with a 10% lidocaine solution, sprayed in the pharyngeal area, combined with a bolus dose of 2% lidocaine, applied to the vocal cords and tracheobronchial tree. EBUS-TBNA was performed using a linear curved-array ultrasonic bronchoscope BF TYPE UC180F (Olympus, Tokyo, Japan). This bronchoscope had an outer diameter of 6.9 mm, a 2.0 mm instrument channel, and 30 degree oblique forward-viewing optics. An electronic convex-array ultrasound transducer was attached at the distal tip of this instrument and covered by a balloon, which was inflatable with water. Scanning allowed for a penetration of 5 cm and was performed at a frequency of 7.5 MHz. Image processing was performed by Aloka ProSound α5sx (Hitachi, Tokyo, Japan). TBNA was performed by passing a dedicated 22- or 21-G needle (ViziShot, Olympus, Tokyo, Japan). A central stylet was passed through the working channel of the bronchoscope, and then advanced through the airway wall, and into the lymph nodes under real-time ultrasound control. An integrated color–power Doppler ultrasound (Olympus, Tokyo, Japan) was used to exclude vessels prior to needle puncture. After stylet removal, suction was applied using a syringe, while manipulating the needle back and forth approximately 10–15 times within the lesion. A minimum of three needle passes per lymph node were performed. After the sampling, suction was released slowly and the needle was retracted. Lymph node aspirates were smeared onto glass slides and air-dried for cytological examination, and put into the separated container and filled with formalin for histological examination of cell blocks. No rapid onsite cytology was performed.

EBUS-TBNA was considered as diagnostic if a clear, definite cytological or histological diagnosis of lung cancer, sarcoidosis, or another disease was obtained (positive specimens), or if lymphocytes and normal lymphoid tissue were found (negative specimens) [13]. It was considered non-diagnostic if neither a definite diagnosis, nor lymphocytes, nor normal lymphoid tissue were found [13]. Final diagnosis was based on EBUS-TBNA, surgical methods, and clinical or radiological surveillance for at least six months [13].

Complications with EBUS-TBNA were classified as major (massive bleeding, heart rhythm disorders requiring treatment, seizures, myocardial infarction, lung edema, pneumothorax requiring drainage or aspiration, sedation overdose requiring ventilation or antidotes, unplanned hospitalization, and death) and minor (minor or moderate bleeding; heart rhythm disorders not requiring treatment; hypotension requiring treatment; and poor tolerance of procedure, which caused termination of the procedure) [16].

The study was approved by the Kaunas Regional Biomedical Research Ethics Committee (No. BE-2-20, issued on 9 February 2015).

### Statistical Analysis

Data were analyzed using the SPSS 16.0 (version for Windows) (IBM corporation, NewYork, NY, USA) statistical software package. Data were presented as frequencies or means ± standard deviation. Categorical data were analyzed using the chi-square (χ^2^) test. For comparisons of data in more than two groups, we used an analysis of variance (ANOVA), and, in two groups, the Student’s *t-*test. Diagnostic sensitivity, specificity, positive predictive value (PPV), negative predictive value (NPV), and accuracy (yield) were calculated using the following standard definitions: sensitivity—the probability of getting a positive test result in a subject with the disease; specificity—the probability of getting a negative test result in a subject without the disease; positive predictive value—the probability of having the disease of interest in a subject with a positive test result; negative predictive value—the probability of not having a disease in a subject with a negative test result; diagnostic accuracy—a proportion of correctly classified subjects among all subjects. The unit of analysis was patients. Values of *p* < 0.05 were considered to indicate statistical significance.

## 3. Results

**Figure 1:**
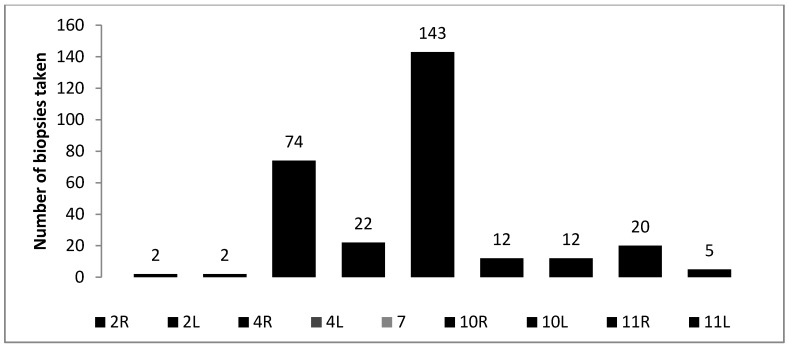
Frequency of biopsied lymph nodes in different stations.

Out of the 215 patients who underwent EBUS-TBNA, 71 (33.1%) were women and 144 (66.9%) were men. The mean age of patients was 58.6 ± 14.9 years (range 21–83). The main reasons for performing EBUS-TBNA were the following: suspected lung cancer, where primary diagnosis was needed (some together with staging) (n = 128, 59.5%); staging of known lung cancer (n = 24, 11.1%); suspected sarcoidosis (n = 35, 16.3%); and mediastinal lymphadenopathy of unknown origin (n = 24, 11.1%). Out of the total296 lymph nodes that were sampled, subcarinal and right paratracheal were most frequently biopsied (Figure 1).

EBUS-TBNA was diagnostic in 176 (81.9%) cases of cytological, 147 (68.4%) cases of histological, and 191 (88.9%) of the combined evaluation. The positive findings were found slightly more often in histologic (cell block) specimens compared with cytology smears, but cytologic specimens had less non-diagnostic results. The highest rate of positive results and lowest rate of non-diagnostic results were found when cytology and histology specimens were combined (Table 1). Among patients undergoing EBUS-TBNA, lung cancer was finally diagnosed in 107 patients (49.8%), sarcoidosis in 52 patients (24.2%), reactive lymph nodes in 38 patients (17.7%), tuberculosis in four patients (1.9%), and metastatic tumors or tumors of unverified location in 3 patients (1.4%). There were 11 patients (5.0%) that were lost in follow up. Patients with a final diagnosis of lung cancer or reactive lymphadenopathy were found to be older than patients with a final diagnosis of sarcoidosis (64 ± 10.7; 60.9 ± 10.9; and 43.4 ± 13.9, respectively, *p* < 0.0001).

For the entire group of patients, the sensitivity of cytology specimens, histology specimens, and the combined results were 65.7%, 70.1%, and 80.7%, respectively, with an NPV of 46.2%, 48.4%, and 60.5%, respectively. As all of the positive cytology and histology specimens were assumed to be true positive, specificity and PPV were 100%. The diagnostic accuracy was 73.5%, 76.4%, and 85.1% for the cytology specimens, histology specimens, and combined results, respectively. The sensitivity and diagnostic accuracy was significantly higher when the cytology and histology specimens were combined, compared with the cytology or histology results that were evaluated separately (*p* < 0.05). Additionally, the sensitivity and diagnostic accuracy of EBUS-TBNA procedures increased significantly over time (Table 2).

The overall diagnostic yield, grouped by final diagnosis, is presented in Table 3. A more detailed analysis of the sensitivity, NPV, and diagnostic accuracy of EBUS-TBNA for the most often diagnosed diseases—lung cancer and sarcoidosis—are presented in Table 4. In lung cancer, the sensitivity of the combined evaluation was significantly higher when compared with the sensitivity of cytology or histology, which were evaluated separately. No such association was found for NPV or diagnostic accuracy. For the sarcoidosis group, the sensitivity increased significantly in the combined evaluation, compared with the cytological evaluation alone (this was not shown, however, when compared to the histological evaluation). The tendency for a higher sensitivity of cytology specimens in the lung cancer group, compared with the sarcoidosis group, was found (*p* = 0.09). Significantly higher NPV was detected for the histological and combined evaluation in the sarcoidosis group, compared with lung cancer (*p* < 0.05). No significant differences in diagnostic accuracy between these two groups were detected. No significant differences in the sensitivity, NPV, and diagnostic accuracy was found when comparing younger and older (>70 years of age) patients (82.4% versus 87.2%, 87.9% versus 80.8%, and 92.3% versus 91.7%, respectively).

While analyzing the most frequently biopsied lymph nodes, the following results were found: In subcarinal lymph nodes, sensitivity, NPV, and diagnostic accuracy were 67.6%, 50.0%, and 75.5%, respectively, for cytology specimens; 67.3%, 49.3%, and 75.2%, respectively, for histology specimens; and 80.6%, 62.5% and 85.3%, respectively, when both specimens were combined. In comparison, sensitivity, NPV, and diagnostic accuracy of the samples from the right paratracheal lymph node were 63.9%, 37.1% and 70.3%, respectively, for cytology specimens; 60.7%, 35.1% and 67.6%, respectively, for histology specimens; and 78.7%, 50.0% and 82.4%, respectively, when both specimens were combined (*p* < 0.05 for combined results compared to cytology or histology separately). As all positive cytology and histology specimens were assumed to be true positive, specificity and PPV were 100%.

There were no complications with EBUS-TBNA in our clinical practice.

## 4. Discussion

We found that the sensitivity of the cytology specimens, histology specimens, and combined results for the entire group of patients was 65.7%, 70.1% and 80.7%, respectively, with a diagnostic accuracy of 73.5%, 76.4% and 85.1%, respectively. The sensitivity and diagnostic accuracy were 84.1% and 92.1%, respectively, for lung cancer, and 78.8% and 94.9%, respectively, for sarcoidosis. Our results in lung cancer seemed to have a lower diagnostic yield compared with the first reported clinical trials of EBUS-TBNA (with reported sensitivities well above 90%). One of the early studies on EBUS-TBNA, performed by Yasufuku et al. [17], on 70 patients with hilar and/or mediastinal lymphadenopathy, visible on chest computed tomography, had a sensitivity of 95.7%. Lee et al. [18] conducted a prospective study on 91 patients with strongly suspected or histologically confirmed non-small cell lung cancer, with a lymph node diameter of 5–20 mm, which was accessible with EBUS-TBNA. They reported a sensitivity of 93.8% and a negative prognostic value of 96.9%. Herth et al. [11] conducted real-time EBUS-TBNA on 502 consecutive patients with suspected lung cancer who showed evidence of mediastinal and hilar lymph node enlargement. The sensitivity and specificity were 94% and 100%, respectively. These findings were partly confirmed in a meta-analysis, where reported sensitivity ranged from 85–100%, and NPV ranged from 11–97.4% in the staging of lung cancer [14]. On the other hand, in the large prospective study of 150 suspected lung cancer patients, conducted by Wallace et al., EBUS-TBNA showed a sensitivity of only 69% [19].

Many factors could have influenced these discrepancies. For example, the majority of these previously reported trials were prospective, well-conducted clinical studies; the patients that were investigated in these trials were usually preselected, with a high prevalence of lung cancer, and referred to large bronchoscopy centers; and EBUS-TBNA was carried out by experienced investigators. These reasons probably led to better results and might not have reflected the real clinical situation. Further studies showed that the value of EBUS-TBNA in daily clinical practice differed from the value in clinical trials. The sensitivity could have depended on the design of the trials, with retrospective studies that indicated a lower diagnostic yield. Agarwal et al. showed that the diagnostic yield for diagnosing sarcoidosis in retrospective studies was only 74.3%, compared with 83.9% in prospective studies [7]. Lange et al. found that, of the cases analyzing a routine clinical practice with unselected patients, the sensitivity of cytology and negative cytology, obtained by EBUS-TBNA, was only 61.4% and 73%, respectively [13].

The efficacy of EBUS-TBNA also seemed to depend on the investigated pathology. The highest sensitivity of EBUS-TBNA was found in the patients with known lung cancer [13]. For example, in a study performed by Anraku et al. [20], where EBUS-TBNA was performed on a patient population with previously treated lung cancer, the sensitivity was 93.1%, specificity 100%, and diagnostic accuracy 95.1%, for this procedure. According to Wada et al., the sensitivity, specificity, and diagnostic accuracy was also shown to be high in the lymph node staging of lung cancer (96.4%, 100%, and 97.2%, respectively) [21]. In the studies where lung cancer was not so prevalent, these numbers differed [22]. Where the study population was more heterogenic, with respect to the disease (tuberculosis, sarcoidosis, and lung cancer), EBUS-TBNA was diagnostic only in 74.5% of patients. Sensitivity, specificity, and negative and positive predictive values were also lower (81.7%, 100%, 22.73% and 100%, respectively) [22].

In diagnosis of sarcoidosis, the value of EBUS-TBNA seemed to be lower than that of lung cancer, with reported diagnostic yields ranging from 54 to 93%, with pooled diagnostic accuracy being 79% [7]. Our results were in line with these findings. The sensitivity and diagnostic accuracy of sarcoidosis were 78.8% and 94.9%.

According to many authors [23,24], the diagnostic value of EBUS-TBNA also relied heavily on the expertise of the bronchoscopist performing the procedure. EBUS-TBNA sample representativeness increased with time and the increased performance of the bronchoscopist. Learning to perform this procedure and maintaining skills required continuous practice. This was shown in our study as well (Table 2). In our clinical practice, the sensitivity of EBUS-TBNA obtained cytology or histology did not reach 90%, but, informativeness of the procedure increased significantly (*p* < 0.05) with increased experience (Table 2).

Another factor that might have influenced the sensitivity of EBUS-TBNA, was the method in which a patient was selected for the procedure. It was reported that sensitivity was higher in the studies that enrolled patients on the basis of a positive computed tomography or positron emission tomography result (according to the meta-analysis: 94%, 95%, and CI of 93 to 96%) [25] compared with those that enrolled patients regardless of the result (76%, 95%, and CI of 65 to 85%) (*p* = 0.02). In our center, patients were selected for EBUS-TBNA after a computed tomography or positron emission tomography–computed tomography scanning.

EBUS-TBNA obtained cytology samples do not always provide sufficient information. Sometimes additional tissue is required, which can be achieved by processing sampled material to cell blocks. This enables histological diagnosis and immunohistochemical staining. With the development of new treatments for lung cancer, which have different degrees of efficacy and toxicity, an accurate pathologic classification has become essential. This can be enabled by cell blocks, which are recommended for routine use during lung cancer diagnosis [26,27]. In our study, in a group with lung cancer, EBUS-TBNA showed sensitivity of 72.9% in cytological samples; 72.9% in histological samples; and 84.1% when results were combined. It can be seen that the addition of histologic samples, made from cell blocks, to cytological smears, increased sensitivity of EBUS-TBNA for the diagnosis of lung cancer. Other studies showed similar tendencies. In a study performed by Sanz-Santos J et al. [28], cell blocks could be prepared from 47.9% aspirates—obtained by EBUS-TBNA from lymph nodes and mediastinal masses—and adequate material for diagnosis was recovered from 37.6% of them. It provided additional information in 26.4% of cases, and raised the overall diagnostic yield of EBUS-TBNA from 72.9 to 80%. In this study, cell blocks allowed for the identification of the sub-type of non-small cell lung cancer, provided that the material was suitable for the epidermal growth factor receptor (EGFR) gene mutation analysis, and allowed for the identification of a mutation. Feller-Kopman et al. [29] compared the cytological samples acquired by EBUS-TBNA with biopsies or surgical samples from a series of 88 patients, discovering that the diagnoses were the same in most patients. It was also noted that the most frequent obstacles in the evaluation of cell blocks of EBUS-TBNA were vascularized lymph nodes, which were more likely to contaminate the samples with blood cells, and lymph nodes or masses containing necrotic material. In these situations, a cytopathologist might not have been able to make a diagnosis from these specimens.

Patients with isolated mediastinal lymphadenopathy (IML) were frequently presented to physicians [30]. In this clinical situation, mediastinoscopy was used as a gold standard. However, in one clinical trial [31], EBUS-TBNA prevented 87% of mediastinoscopies. The sensitivity and negative predictive value of EBUS-TBNA in patients with IML were 92% and 40%, respectively [31]. EBUS-TBNA was considered to be a safe, highly sensitive, and cost-saving (when compared to mediastinoscopy) initial investigation in patients with IML. There were no major complications associated with EBUS-TBNA in our study, there were also very few reviewed in the literature. Most of the studies reported no complications of the procedure [32,33,34]. According to a meta-analysis performed by Gu et al., out of 11 reviewed studies and 1299 participants, only two had complications (1 had pneumothorax, 1 suffered from hypoxemia and recovered soon after the procedure) [25].

EBUS-TBNA allowed for the ability to obtain both cytological and histological samples via either a 22-G needle or the more recent 21-G needle. In our study, we used a 22-G needle. Some studies, which compared the samples that were obtained with both needles, did not demonstrate a difference in diagnostic yield within the groups [35]. However, other studies suggested that there was a better preservation of histological structure and an increased number of cells within specimens from the 21-G needle for malignant disease [36]. Additionally, they suggested that the 21-G EBUS-TBNA needle was superior to the 22-G needle for characterizing benign and malignant mediastinal lymphadenopathy [34].

## 5. Conclusions

EBUS-TBNA had high diagnostic yield and was safe in the diagnosis of lung cancer and sarcoidosis. The informative value of EBUS-TBNA histology increased with our experience. It is the main diagnostic standard in our daily clinical practice, and surgical methods are only used as an alternative.

## Figures and Tables

**Table 1:** Positive, negative, and non-diagnostic results of endobronchial ultrasound-guided transbronchial needle aspiration (EBUS-TBNA) for cytology smears specimens, histology specimens obtained through cell blocks, and the combined evaluation.

	Cytology No (%)	Histology No (%)	Combined Evaluation No (%)
Positive	109 (50.7)	115 (54.8)	134 (62.3)
Negative	67 (31.2)	32 (15.2)	57 (26.5)
Non-diagnostic	39 (18.1)	63 (30)	24 (11.2)
Total	215 (100)	210 (100)	215 (100)

**Table 2:** Sensitivity, negative predictive value (NPV), accuracy, positive predictive value (PPV), and specificity of EBUS-TBNA combined results over time.

Year	Sensitivity (%)	NPV (%)	Accuracy (%)	PPV (%)	Specificity (%)
2009	68.8	56.5	77.8	100	100
2010	69.4	57.7	78.4	100	100
2011	95.5	66.7	95.8	100	100
2012	89.3	80.0	92.5	100	100
2013–2014	85.4	62.0	87.3	100	100

*p* < 0.05 for EBUS-TBNA sensitivity and accuracy in years 2009–2010 (combined) compared to years 2011–2014 (combined).

**Table 3:** Positive EBUS-TBNA results by final diagnosis.

	Cytology No (%)	Histology No (%)	Combined Evaluation No (%)
Lung cancer	78 (72.9)	78 (72.9)	90 (84.1)
Metastatic cancer	1 (33.3)	2 (66.7)	2 (66.7)
Sarcoidosis	29 (55.8)	34 (65.4)	41 (78.8)
Tuberculosis	1 (25)	1 (25)	1 (25)
Reactive lymphadenopathy	32 (84.2)	20 (52.6)	33 (86.8)
Total	141 (69.1)	135 (67.8)	167 (81.9)

**Table 4:** Sensitivity, negative predictive value (NPV), diagnostic accuracy, specificity, and positive predictive value (PPV) of cytology, histology, or both, obtained through EBUS-TBNA for lung cancer and sarcoidosis patients.

Results of EBUS-TBNA	Lung Cancer (%)	Sarcoidosis (%)	*p*
Cytology			
Sensitivity	72.9	55.8	0.09
NPV	78.8	87.6	0.053
Accuracy	86.5	89.3	NS
Specificity	100	100	NS
PPV	100	100	NS
Histology			
Sensitivity	72.9	65.4	NS
NPV	79.3	89.9	0.01
Accuracy	86.5	91.6	NS
Specificity	100	100	NS
PPV	100	100	NS
Cytology and/or Histology			
Sensitivity	84.1 *	78.8 **	NS
NPV	86.4	93.7	0.04
Accuracy	92.1	94.9	NS
Specificity	100	100	NS
PPV	100	100	NS

* *p* = 0.038 compared to cytological or histological evaluation. ** *p* = 0.04 compared to cytological evaluation, NS—not significant.
